# IKBKE regulates cell proliferation and epithelial–mesenchymal transition of human malignant glioma via the Hippo pathway

**DOI:** 10.18632/oncotarget.17738

**Published:** 2017-05-10

**Authors:** Jie Lu, Yi Yang, Gaochao Guo, Yang Liu, Zhimeng Zhang, Shicai Dong, Yang Nan, Zhenyi Zhao, Yue Zhong, Qiang Huang

**Affiliations:** ^1^ Department of Neurosurgery, Tianjin Medical University General Hospital, Heping District, Tianjin 300052, China; ^2^ Department of Neurosurgery, Tianjin Baodi People's Hospital, Baodi District, Tianjin 301800, China

**Keywords:** IKBKE, epithelial–mesenchymal transition (EMT), glioma, Hippo pathway

## Abstract

IKBKE is increased in several types of cancers and is associated with tumour malignancy. In this study, we confirmed that IKBKE promoted glioma proliferation, migration and invasion *in vitro*. Then, we further discovered that IKBKE increased Yes-associated protein 1 (YAP1) and TEA domain family member 2 (TEAD2), two important Hippo pathway downstream factors, to induce an epithelial–mesenchymal transition (EMT), thus contributing to tumour invasion and metastasis. We also testified that YAP1 and TEAD2 promoted epithelial–mesenchymal transition (EMT) in malignant glioma. Furthermore, we constructed nude mouse subcutaneous and intracranial models to verify that IKBKE could attenuate U87-MG tumourigenicity *in vivo*. Collectively, our results suggest that IKBKE plays a pivotal role in regulating cell proliferation, invasion and epithelial–mesenchymal transition of malignant glioma cells *in vitro* and *in vivo* by impacting on the Hippo pathway. Therefore, targeting IKBKE may become a new strategy to treat malignant glioma.

## INTRODUCTION

Glioma is the most common primary intracranial tumour, accounting for approximately 46% of cerebral tumours [1]. Glioma has a morbidity of 4.623/100000 and glioblastoma has a morbidity of 2.102/100000 [2]. Although standard therapy including tumour resection and radiotherapy with concomitant and adjuvant temozolomide remains in practice, the median survival time of glioblastoma is still 12–15 months [3, 4]. Malignant glioma has become an intractable disease in neurosurgery, so it is a challenge to define its molecular mechanisms and develop new treatment strategies.

IKBKE, also called IKKε or IKKi, is a non-canonical member of the IKK family containing a series of serine/threonine kinases that also include IKKα, IKKβ, TBK1 and the adaptor protein IKKγ [5–7]. Boehm et al. [8] demonstrated that IKBKE is a new oncogene that is amplified in approximately 30% of breast carcinomas using integrative genomic approaches. Inhibiting IKBKE can promote breast cancer cell apoptosis and cell transformation through activation of the NF-κB pathway. Recent studies have shown that IKBKE is also overexpressed in ovarian cancer [9], endometrial carcinoma [10], prostate cancer [11], glioma [12, 13], renal clear cell carcinoma [14] and non-small cell lung cancer [15, 16]. IKBKE is also closely related to cancer grade in ovarian cancer [9], glioma [12], and lung squamous cell cancer [15]. It also induces tumour chemoresistance in non-small cell lung cancer [16] and ovarian cancer [9]. This evidence indicates that IKBKE is an important factor in carcinogenesis and that IKBKE could become a new therapeutic target in malignancy, but its exact mechanisms of carcinogenesis have not yet been determined.

Epithelial–mesenchymal transition (EMT) is a reversible biological process involved in embryogenesis, tissue regeneration and cancer progression [16]. In primary tumours, the decreased epithelial markers such as E-cadherin, occludin, cytokeratin and the increased mesenchymal markers including N-cadherin, vimentin, snail, slug can promote the tumour invasion and metastasis. Several pathways have been discovered to regulate EMT, including NF-κB, Wnt, PI3k/Akt and so on [17]. More and more researches have revealed that the Hippo pathway has a critical role in EMT.

The Hippo pathway regulates cell proliferation, apoptosis, differentiation and stemness in response to changes of the intracellular and extracellular microenvironment, including cell contact, cell polarity, mechanotransduction and G-protein-coupled receptor (GPCR) signalling [18, 19]. Recent studies have shown that the Hippo pathway is heavily involved in the facilitation of tumourigenesis [20, 21]. The deregulation of the Hippo pathway has been reported frequently in a broad range of cancers, including non-small-cell lung cancer (NSCLC), [22] breast carcinoma [23], hepatocarcinoma [24], and glioma [25]. Orr BA et al [26] reported that YAP1 expression was increased in gliomas, especially for high-grade gliomas and inhibition of YAP1 could suppress glioblastoma proliferation. Briefly speaking, inhibition of the Hippo pathway leading to YAP/TAZ/TEAD hyperactivation accelerated tumour development and promoted the tumour malignancy.

In this article, we demonstrated that IKBKE is overexpressed in several glioma cell lines and can promote glioma cell proliferation, migration and invasion. What's more, IKBKE promotes EMT through enhancing the expressions of YAP1 and TEAD2. Silencing IKBKE can inhibit glioblastoma progression *in vitro* and *in vivo*. These results show that IKBKE contributes to malignant glioma tumourigenesis.

## RESULTS

### Silencing IKBKE inhibits malignant glioma cells proliferation, migration and invasion

**Figure 1:**
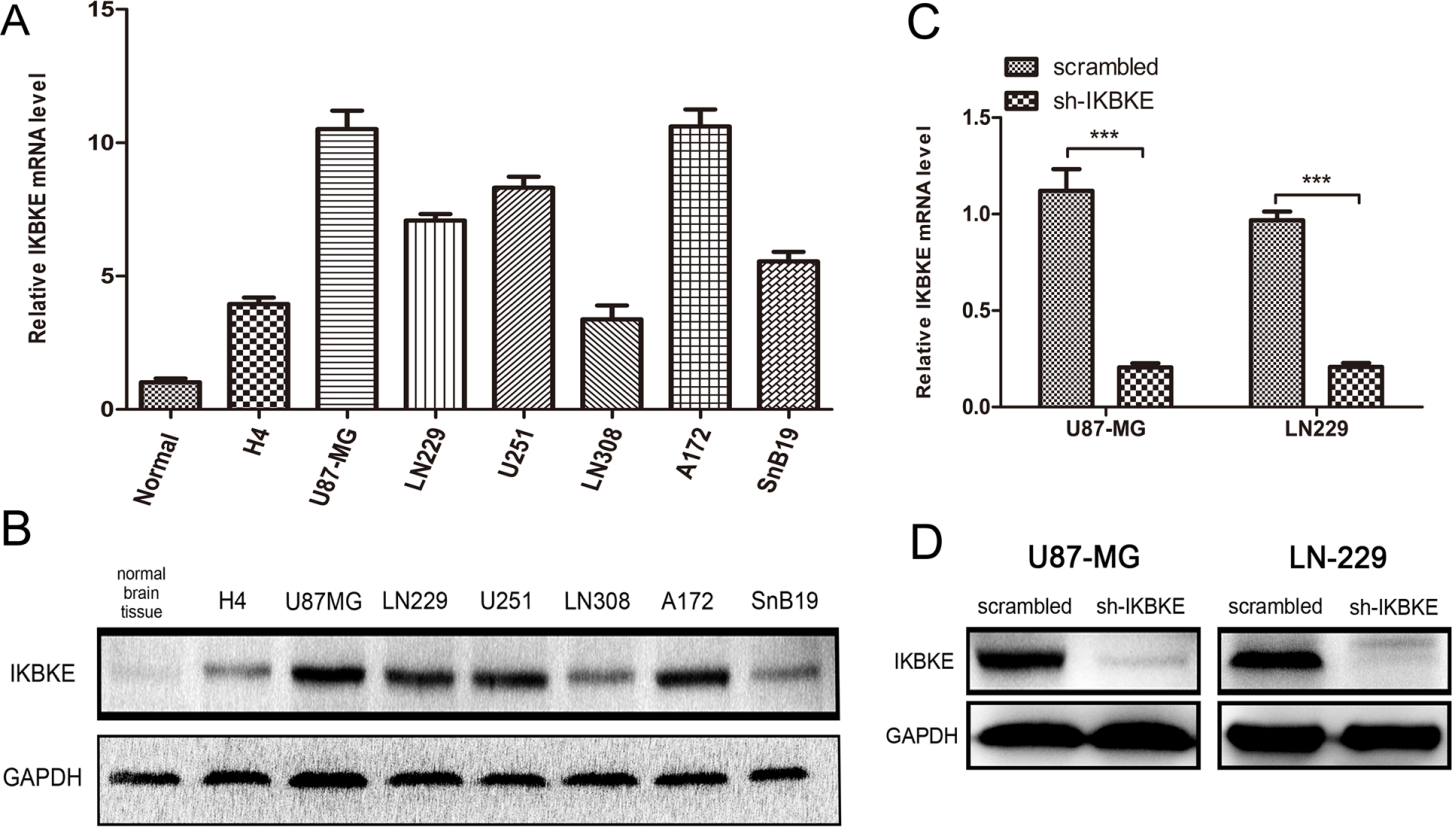
Expression of IKBKE in cell lines and silence of IKBKE using shRNA in U87-MG and LN-229 (**A**, **B**), Expression of IKBKE in normal brain tissue and glioma cell lines detected by real-time RT-PCR and western blot. (**C**, **D**) Silence of IKBKE in U87-MG and LN229 detected by real-time RT-PCR and western blot. (*p < 0.05; **p < 0.01; ***p < 0.001)

We first investigated IKBKE expression levels in normal brain tissue, a low-grade malignant glioma cell line (H4) and high-grade malignant glioma cell lines (U87-MG, LN229, U251, LN308, A172 and SnB19) using real-time RT-PCR (Figure 1A) and western blotting (Figure 1B). We selected U87-MG and LN-229 as representatively investigative cells. The effects from IKBKE prohibition in U87-MG and LN-229 cells transfected with scrambled vector or IKBKE-shRNA lentivirus were confirmed by real-time RT-PCR (Figure 1C) and western blotting (Figure 1D). These data showed that the effect of silencing IKBKE was remarkably evident.

**Figure 2:**
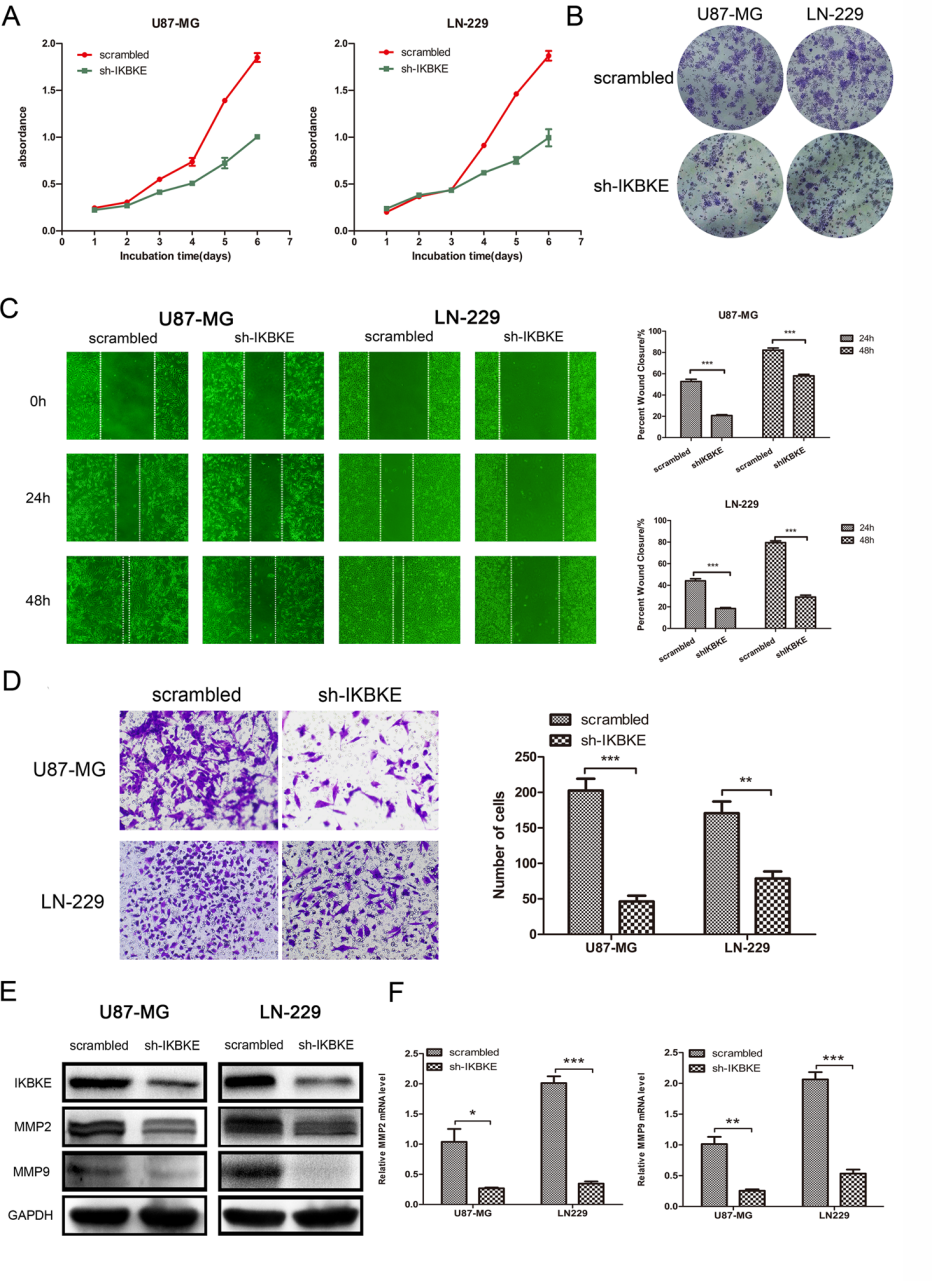
Knockdown of IKBKE inhibited glioma cell proliferation, migration and invasion (**A**) U87-MG and LN229 proliferation was measured by CCK-8 in 6 days (*n* = 5). |(**B**) Clone formation assay in U87-MG and LN229 transfected with IKBKE-shRNA or scrambled control. (**C**) Wound healing assay was used to evaluate cell migration ability after knockdown IKBKE at 24 and 48 hours. (**D**) Glioma cell invasion was assessed by transwell assay. (**E**, **F**) Two important migration and invasion markers (MMP2, MMP9) were detected by western blot and real-time RT-PCR after knocking down IKBKE. GAPDH was used as a positive control. (*p < 0.05; **p < 0.01; ***p < 0.001)

We used the CCK-8 assay to evaluate the different proliferation abilities of U87-MG and LN-229 in knockdown IKBKE and scrambled control cells. The CCK-8 assay (Figure 2A) showed that U87-MG and LN-229 proliferation capacities were significantly impaired as time passed after knockdown IKBKE. What's more, cells treated with IKBKE-shRNA exhibited decreased clone formation and smaller clone diameter compared with scrambled group (Figure 2B). To test whether the downregulation of IKBKE in U87-MG and LN-229 cells affected their migration abilities, we adopted the wound healing assay. The results (Figure 2C) demonstrated that silencing IKBKE inhibited cells' wound healing compared to cells transfected with scrambled lentivirus, suggesting poor migration ability in IKBKE knockdown samples. Meanwhile, a transwell assay was applied to assess cell invasion ability. The experimental results (Figure 2D) demonstrated that the average number of cells with IKBKE-shRNA across chambers was decreased significantly compared to cells with scrambled group, indicating that IKBKE plays an important role in glioma cell invasion. To further elucidate the detailed mechanism, we assessed the protein level changes of MMP2 and MMP9 by real-time RT-PCR and western blot. Western blot (Figure 2E) and real-time RT-PCR (Figure 2F) both showed significant decreases of MMP2 and MMP9 in mRNA and protein level after knocking down IKBKE. The above-mentioned data all imply that IKBKE has a critical effect on malignant glioma cell proliferation, migration and invasion.

### IKBKE promotes epithelial-mesenchymal transition (EMT) through effects on the Hippo pathway

**Figure 3:**
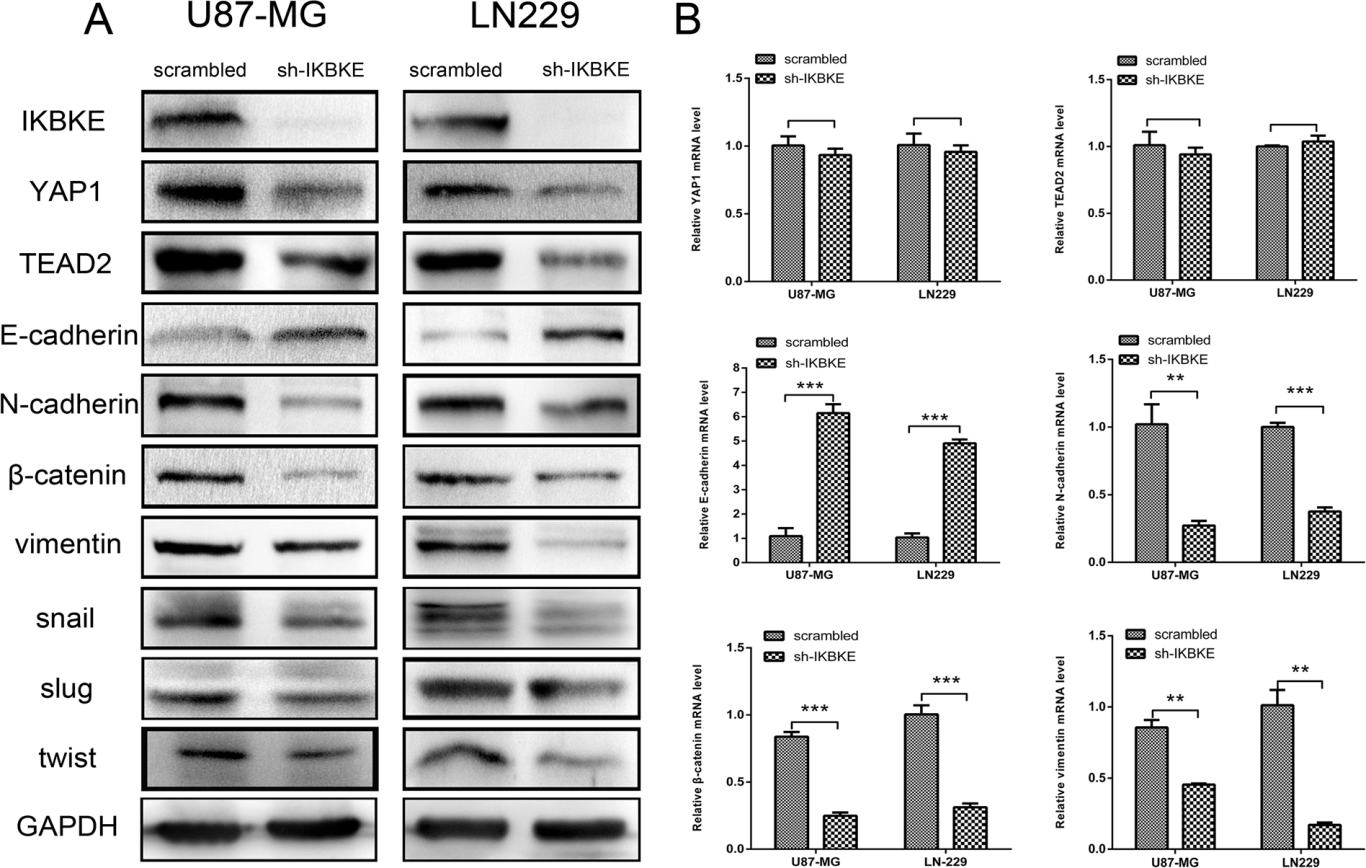
Downregulation of IKBKE reversed epithelial–mesenchymal transition (EMT) via the Hippo pathway (**A**) Decreased protein levels of YAP1, TEAD2 (Hippo pathway downstream factors) and mesenchymal markers (N-cadherin, β-catenin, vimentin, snail, slug, twist), increased protein level of epithelial marker (E-cadherin) were measured by western blot after knockdown IKBKE. GAPDH was used as a positive control. (**B**) mRNA levels of YAP1, TEAD2, E-cadherin, N-cadherin, β-catenin and vimentin were measured by real-time RT-PCR. (*p < 0.05; **p < 0.01; ***p < 0.001)

Epithelial–mesenchymal transition is a vital process in malignant tumour invasion and metastasis. To investigate whether IKBKE expression level impacts EMT markers in U87-MG and LN-229 cells, real-time RT-PCR, western blot and immunofluorescence techniques were used for analysis. Compared to scrambled vector cells, the epithelial marker E-cadherin level increased (Figure 3A), while mesenchymal markers N-cadherin, vimentin, Snail, Slug and twist levels decreased (Figure 3A) in IKBKE- shRNA cells according to western blot. Additionally, YAP1 and TEAD2, two vital Hippo pathway downstream transcription factors, obviously decreased in IKBKE-silenced tumour cells (Figure 3A), suggesting that the reversed EMT process caused by IKBKE downregulation was likely regulated by the Hippo pathway. Meanwhile, knocking down IKBKE resulted in the upregulation of E-cadherin mRNA expression (Figure 3B) and downregulation of N-cadherin, β-catenin, vimentin mRNA expression (Figure 3B), suggesting that IKBKE could influence EMT on the mRNA level. While YAP1 and TEAD2 mRNA levels remained unchanged after knocking down IKBKE, showing that IKBKE likely influenced these markers via posttranslational modification rather than translational regulation. Similarly, E-cadherin, β-catenin, vimentin and snail level demonstrated immunofluorescence (Supplementary Figure 1) changes according to western blot analysis. As for β-catenin, vimentin and snail, fluorescence intensities of IKBKE-shRNA-transfected glioma cells were lower than those infected scrambled vector. In contrast, the intensity of E-cadherin was reversed.

**Figure 4:**
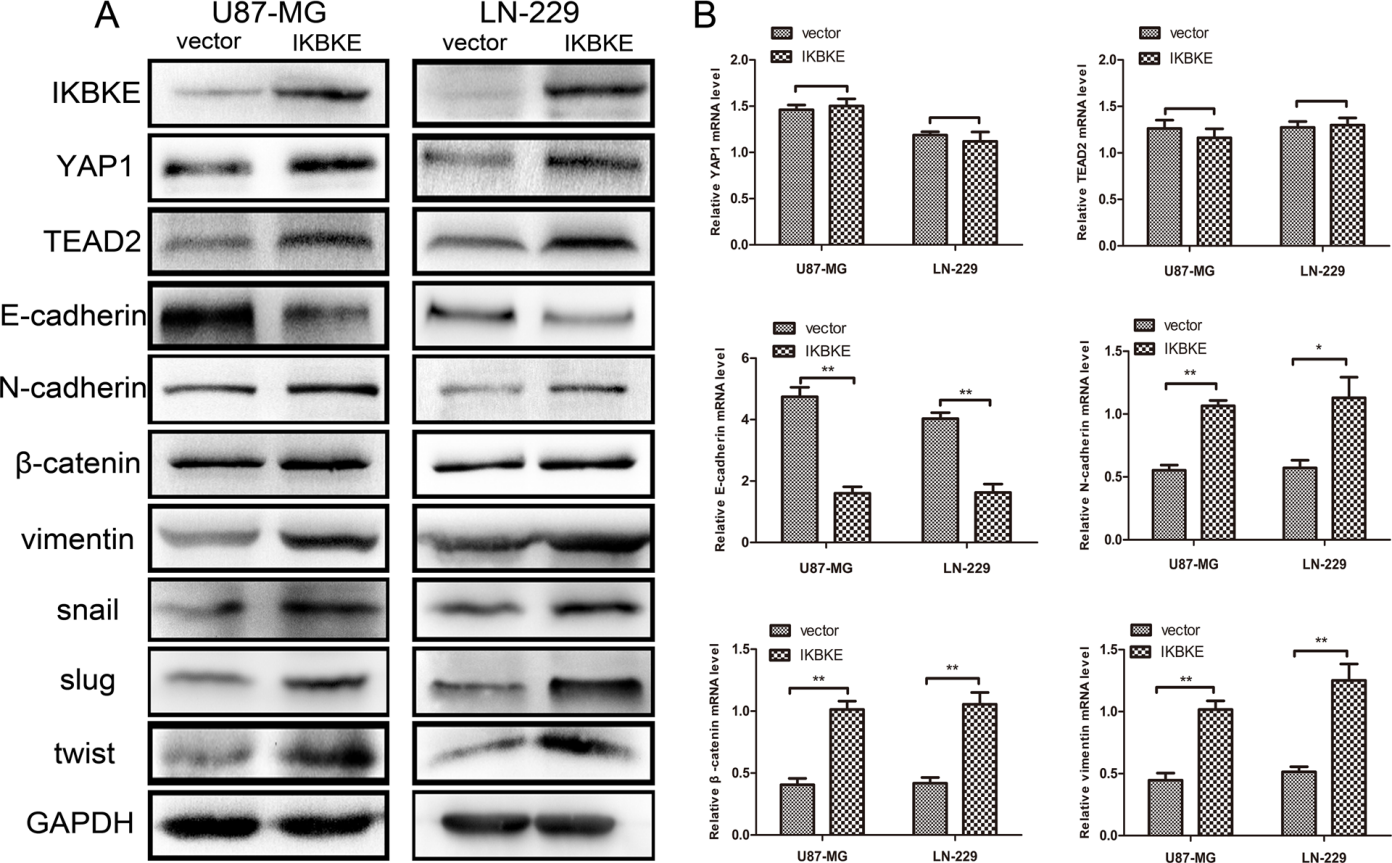
Overexpression of IKBKE promoted epithelial–mesenchymal transition (EMT) via the Hippo pathway (**A**) Increased protein levels of YAP1, TEAD2 (Hippo pathway downstream factors) and mesenchymal markers (N-cadherin, β-catenin, vimentin, snail, slug, twist), decreased protein level of epithelial marker (E-cadherin) were measured by western blot after overexpressing IKBKE. GAPDH was used as a positive control. (**B**) mRNA levels of YAP1, TEAD2, E-cadherin, N-cadherin, β-catenin and vimentin were measured by real-time RT-PCR. (*p < 0.05; **p < 0.01; ***p < 0.001)

What's more, we overexpressed IKBKE and then testified YAP1, TEAD2, and EMT markers expression. As shown in Figure 4A, the EMT markers N-cadherin, β-catenin, vimentin, Snail, Slug and twist levels were increased and E-cadherin expression was decreased after overexpressing IKBKE. Meanwhile, the expression levels of YAP1 and TEAD2 were also increased after overexpressing IKBKE compared to vector group, showing that IKBKE increased EMT process probably via the Hippo pathway. Next, we detected that the E-cadherin mRNA expression was decreased while N-cadherin, β-catenin, vimentin mRNA expression was elevated after overexpressing IKBKE compared to vector group (Figure 4B). In addition, the mRNA expression of YAP1 and TEAD2 was negligibly changed (Figure 4B), further indicating that IKBKE maybe influenced YAP1 and TEAD2 via posttranslational modification. All of the above outcomes clarified that IKBKE could promote EMT and the expression of YAP1, TEAD2 in glioblastoma.

### YAP1 and TEAD2 promotes epithelial-mesenchymal transition (EMT)

**Figure 5:**
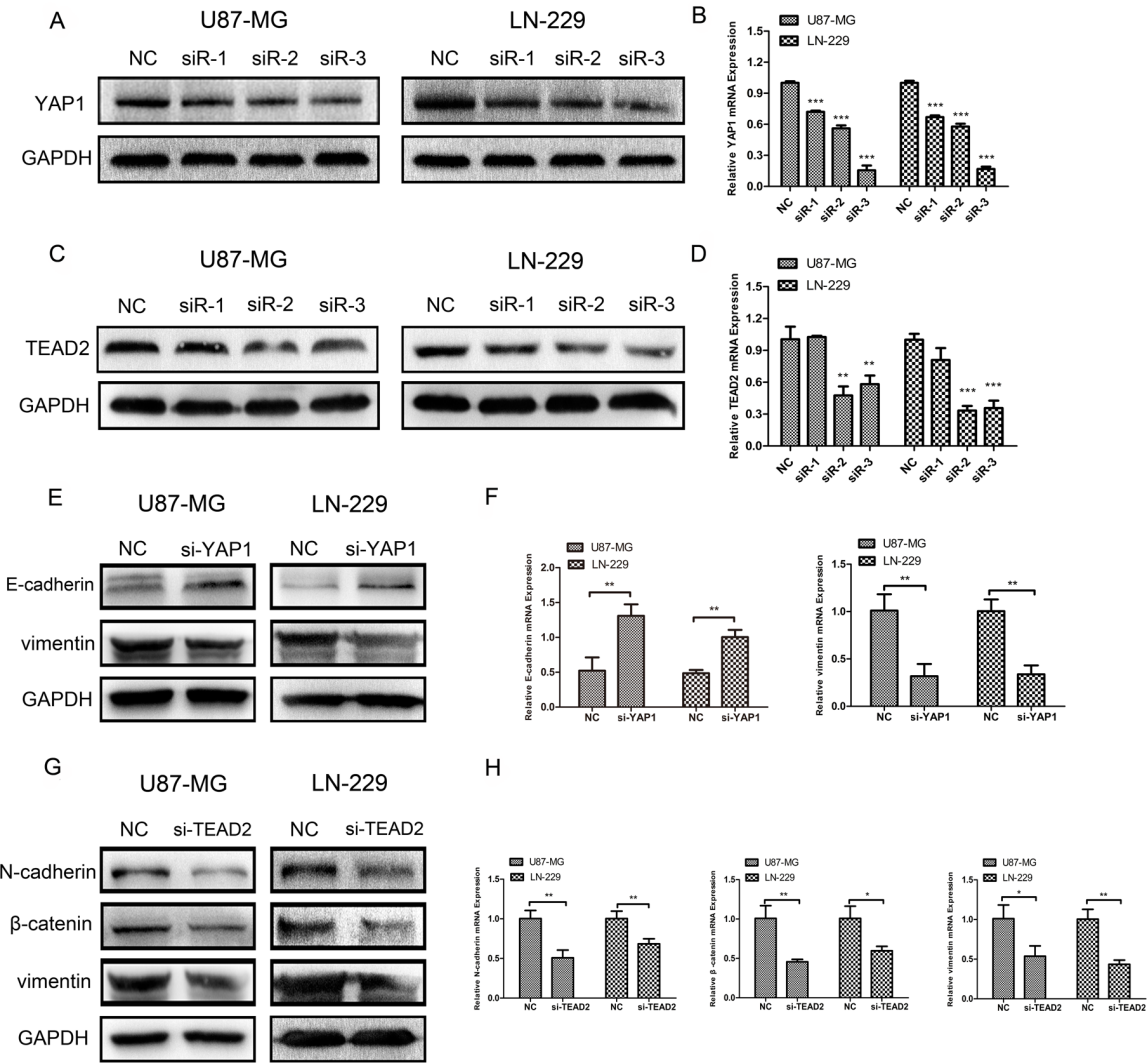
Silencing YAP1 and TEAD2 could inhibit epithelial-mesenchymal transition (EMT) respectively (**A**, **B**) YAP1 expression in U87-MG and LN-229 treated with siRNA-NC and siRNA1–3 was measured by western blot and real-time RT-PCR. (**C**, **D**) TEAD2 expression in U87-MG and LN-229 treated with siRNA-NC and siRNA1–3 was determined by western blot analysis and real-time RT-PCR. (**E**, **F**) Protein expressions and mRNA levels of E-cadherin and vimentin were measured by western blot and real-time PR-PCR after U87-MG and LN-229 were transfected with YAP1-siRNA3. (**G**, **H**) Protein expressions and mRNA levels of N-cadherin, β-catenin and vimentin were measured by western blot and real-time RT-PCR after glioma cell lines were transfected with TEAD2-siRNA2. (*p < 0.05; **p < 0.01; ***p < 0.001)

To further explore whether the Hippo pathway could influence epithelial-mesenchymal transition (EMT) in glioma cell lines, we first designed siRNA to respectively downregulate the expression of YAP1 and TEAD2, two important downstream factors of the Hippo pathway. After transfection with YAP1-siRNA in U87-MG and LN-229, we confirmed the effect of YAP1 downregulation using western blot analysis (Figure 5A) and real-time RT-PCR (Figure 5B). Similarly, the results of knocking down TEAD2 in U87-MG and LN-229 using siRNA were ascertained by western blot (Figure 5C) and real-time RT-PCR (Figure 5D). We chose siRNA-3 of YAP1-siRNA and siRNA-2 of TEAD2-siRNA in which the most efficient knockdown was observed for the following experiment. After silencing YAP1 by siRNA-3 in U87-MG and LN-229, the expression level of vimentin was decreased according to western blot (Figure 5E) and the mRNA expression of vimentin was correspondingly decreased according to real-time RT-PCR (Figure 5F). The expression level of E-cadherin was increased using western blot (Figure 5E) and the mRNA expression of E-cadherin was increased as well (Figure 5F). Furthermore, we discovered that the expression of N-cadherin, β-catenin, and vimentin was decreased using western blot analysis (Figure 5G), and the mRNA levels in N-cadherin, β-catenin, vimentin were significantly reduced as found by real-time RT-PCR (Figure 5H) after knocking down TEAD2 expression.

**Figure 6:**
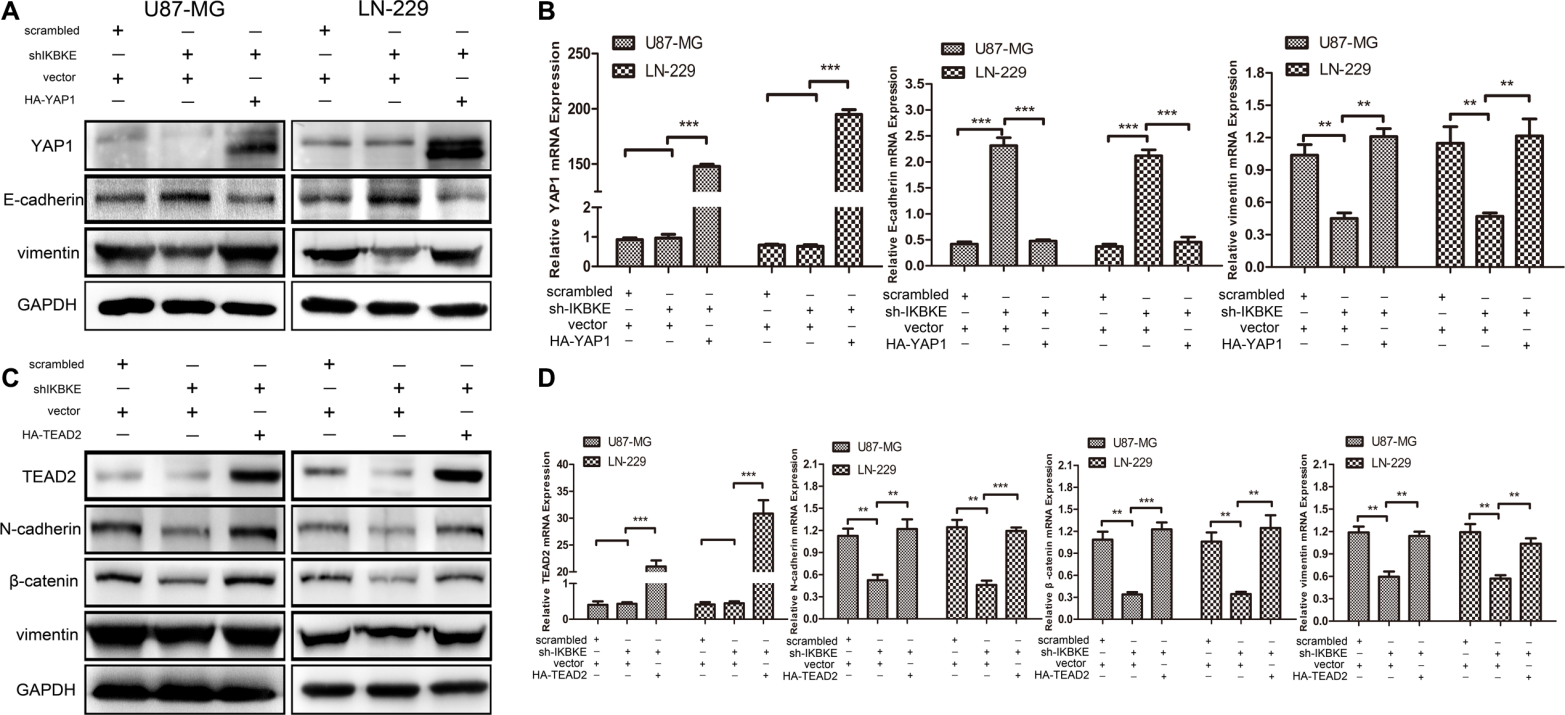
Overexpression of YAP1 and TEAD2 recovered the EMT after cells treated with IKBKE-shRNA (**A**, **B**) The expressions of YAP1, E-cadherin and vimentin was analysized by western blot and real-time RT-PCR overexpressing YAP1 in IKBKE-shRNA-treated cells. (**C**, **D**) The expressions of TEAD2, N-cadherin, β-catenin and vimentin were analysized by western blot and real-time RT-PCR after overexpressing TEAD2 in IKBKE-shRNA-treated cells. (*p < 0.05; **p < 0.01; ***p < 0.001)

Additionally, we respectively overexpress YAP1 and TEAD2 in glioblastoma cell lines transfected with IKBKE-shRNA. As shown in Figure 6A, the E-cadherin expression was first increased after transfection with IKBKE-shRNA and then decreased as YAP1 was overexpressed. However, the vimentin expression was decreased when transfected with IKBKE-shRNA and then increased as YAP1 was overexpressed. Similarly, the expression levels of N-cadherin, β-catenin and vimentin were recovered after overexpressing TEAD2 (Figure 6C). What's more, the E-cadherin, vimentin mRNA expression after overexpression YAP1 (Figure 6B) and N-cadherin, β-catenin, vimentin mRNA expression after overexpression TEAD2 (Figure 6D) were altered as protein expression. These data demonstrated that YAP1/TEAD2, the Hippo pathway downstream transcription factors, could promote the epithelial-mesenchymal transition (EMT) in malignant glioma cells and further explained that IKBKE enhanced epithelial-mesenchymal transition (EMT) via the Hippo pathway.

### IKBKE directly interacts with YAP1 and TEAD2

**Figure 7:**
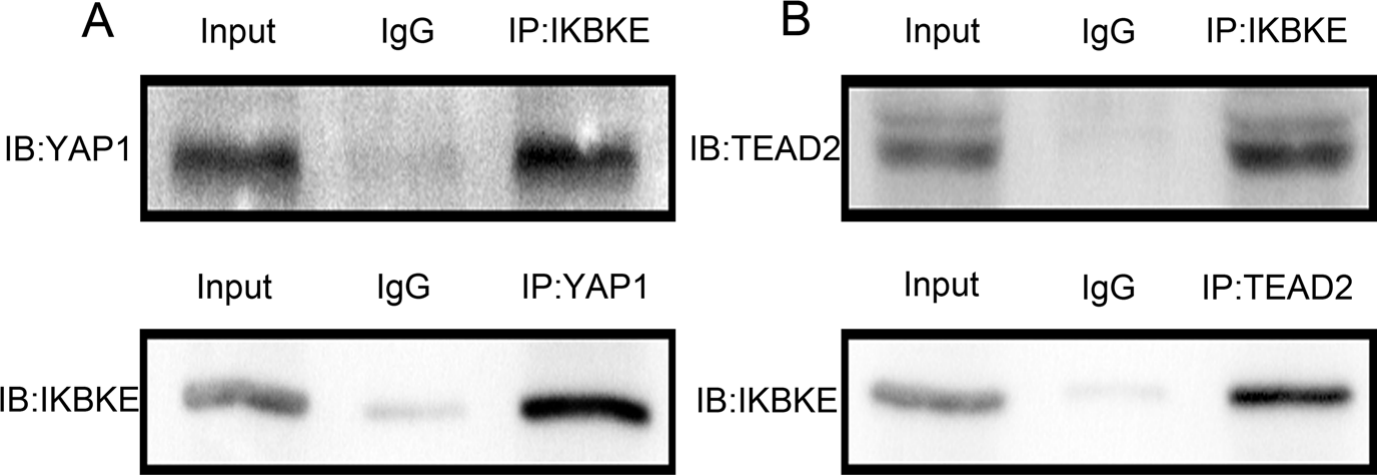
IKBKE could directly interact with YAP1, TEAD2 (**A**) IKBKE bound to YAP1 using co-IP. (**B**) IKBKE combined with TEAD2 using co-IP.

As mentioned above, IKBKE promoted YAP1 and TEAD2 protein expression but little impact on mRNA expression. We investigated detailed effects between IKBKE and YAP1, TEAD2. We firstly used U87-MG extracts to immunoprecipitate IKBKE and detected YAP1 by immunoblot. Then immunoprecipitate YAP1 and detected IKBKE by immunoblot (Figure 7A), showing that IKBKE could combine with YAP1. Similarly, we next certified that IKBKE also directly interacted with TEAD2 (Figure 7B) using endogenous co-immunoprecipitation (co-IP). These data demonstrated that IKBKE directly interacted with YAP1 and TEAD2.

### Knockdown of IKBKE inhibits tumour formation in mouse subcutaneous and intracranial models

**Figure 8:**
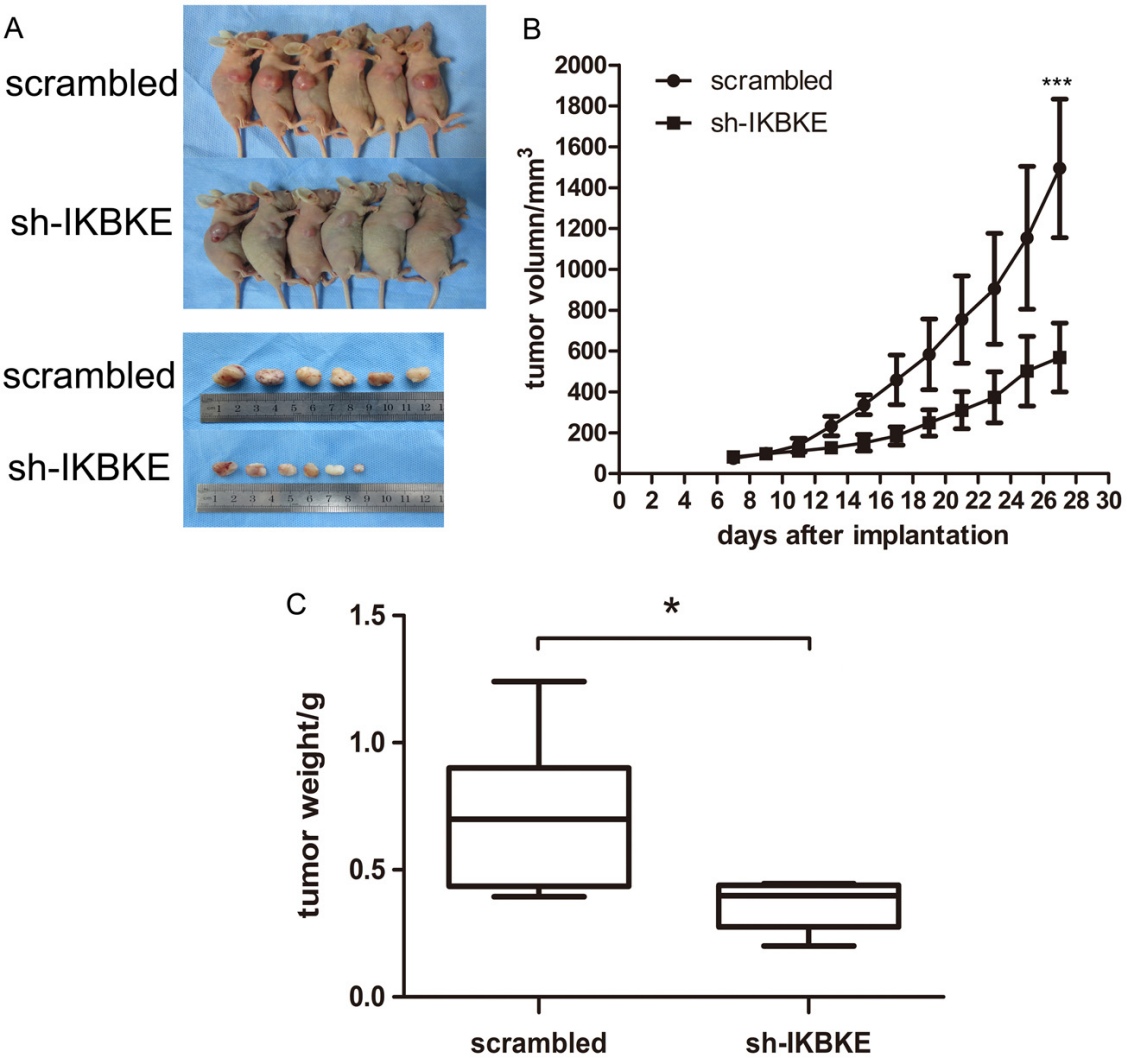
Downregulation of IKBKE inhibited tumourigenesis in subcutaneous nude mice (**A**) Tumours in nude mouse treated with IKBKE-shRNA were smaller than those treated with transfected scrambled vector. (**B**) Tumour growth in IKBKE-shRNA nude mice is slower than that in scrambled vector mice. (**C**) Tumour weight compared between two groups of mice infected with IKBKE-shRNA and scrambled vector. (*p < 0.05; **p < 0.01; ***p < 0.001)

**Figure 9:**
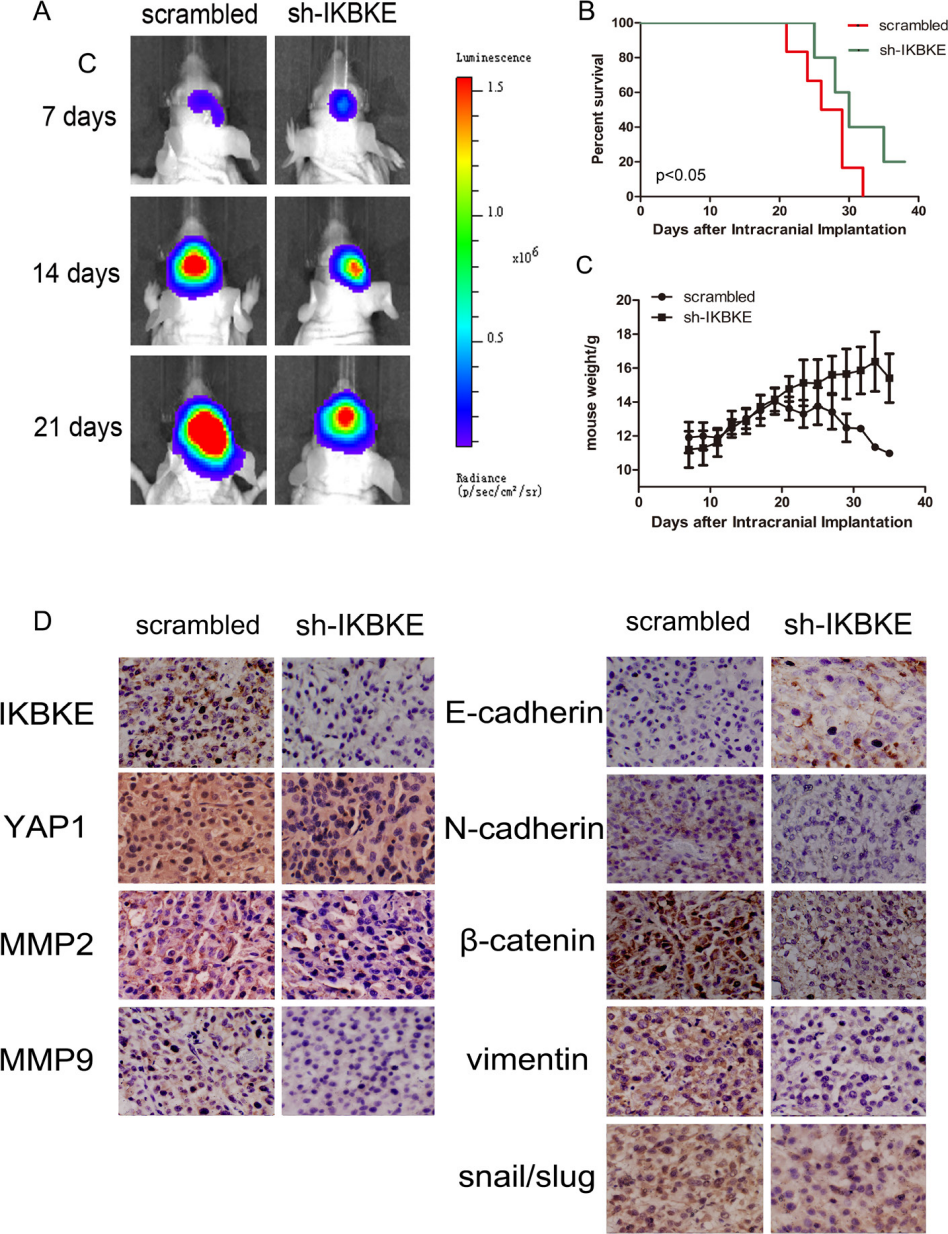
Downregulation of IKBKE inhibited tumourigenesis in intracranial nude mouse (**A**) Tumour size was determined by luminescence imaging. (**B**) A survival curve was used to detect differences in mouse survival times between the two groups. (**C**) Mouse weight was recorded as a measure of mouse nutrition in the two groups. (**D**) Immunohistochemistry analysis of the expression of IKBKE, YAP1, MMP2, MMP9, E-cadherin, N-cadherin, β-catenin, vimentin and snail in IKBKE-shRNA-treated tumours compared to tumours in the scrambled group. (*p < 0.05; **p < 0.01; ***p < 0.001)

Given that IKBKE knockdown could inhibit the tumourigenicity of glioma cells *in vitro*. We further investigated U87-MG tumourigenesis *in vivo*. First, 6 nude mice were subcutaneously inoculated with scrambled vector U87-MG cells, while another 6 mice received IKBKE-knocked down U87-MG cells. The tumour volume was monitored every 2 days and all mice were sacrificed to obtain the implanted tumour weight. Tumour size, growth and weight from mice infected with scrambled lentivirus demonstrated statistical increases over the tumours from mice transfected with IKBKE-shRNA as determined via data analysis (Figure 8A–8C). Then, we respectively implanted scrambled/luciferase lentivirus and IKBKE-shRNA/luciferase lentivirus U87-MG cells into nude mouse cerebrums. Images of intracranial tumour size were taken on the 7th, 14th and 21st days after orthotopic xenotransplantation. As shown in Figure 9A, intracranial tumours transfected with IKBKE-shRNA were significantly smaller than those infected with scrambled lentivirus. Likewise, IKBKE downregulation was associated with a longer survival rate (Figure 9B) and a decreased loss of weight in mice (Figure 9C). Furthermore, we stained the intracranial tumours with IKBKE, YAP1, MMP-2, MMP-9, E-cadherin, N-cadherin, β-catenin, vimentin, and snail. Immunohistochemistry showed increased expression of E-cadherin and decreased expressions of MMP-2, MMP-9, N-cadherin, β-catenin, vimentin and snail, a result consistent with *in vitro* experiments (Figure 9D). Interestingly, YAP1 expression in the glioma cell nucleus transfected with IKBKE-shRNA was reduced much more than in the cytoplasm. Altogether, these findings showed that knockdown of IKBKE inhibited the tumourigenesis of malignant glioma *in vivo*.

## DISCUSSION

Although it is still a controversial discussion, whether epithelial–mesenchymal transition (EMT) has a real association with malignant glioma invasion, considering that glial cells' origins, features and behaviours differ from classical epithelium, is the basis for more future studies highlighting numerous EMT markers that play a significant role in glioma malignancy. Myung JK et al [27] showed that snail promoted glioma cell proliferation, migration, and invasion by promoting EMT induction *in vitro*. Yang HW et al [28] demonstrated that slug accelerated glioma cell invasion *in vitro* and promoted angiogenesis and glioblastoma growth *in vivo*. Additionally, Mikheeva SA et al [29] demonstrated that TWIST1 enhanced GBM invasion in concert with mesenchymal change. Therefore, the term “glial-to-mesenchymal transition (GMT)” or EMT-like process, a substitute of epithelial–mesenchymal transition (EMT) in glioma, has been increasingly proposed [18, 30].

In this study, we primarily confirmed that IKBKE downregulation could inhibit glioma cell abilities including proliferation, migration and invasion *in vitro*. Subsequently, the changes in a series of EMT markers were detected by western blot, real-time RT-PCR after knocking down and overexpressing IKBKE, indicating that IKBKE promote epithelial-mesenchymal transition. Next, we confirmed that YAP1 and TEAD2 could promote EMT by western blot and real-time PCR, further emphasizing that IKBKE could influence EMT via the Hippo pathway in U87-MG and LN-229. Then, we verified that knockdown of IKBKE inhibits tumourigenesis *in vivo* by creating nude mouse subcutaneous and intracranial models, thus illustrating that IKBKE, as an oncoprotein, played a crucial role in malignant glioma progression.

Our research also discovered that IKBKE directly interacted with YAP1 and TEAD2 (Figure 7A, 7B) and IKBKE up-regulated YAP1 and TEAD2 expression. It has been well-established that YAP1 activation is crucial for glioma cell invasion and proliferation [26]. So we assumed that IKBKE promoted malignant glioma growth and EMT via enhancing the expression of YAP1 and TEAD2. Recent studies have increasingly confirmed our points of view. Pei et al [31] demonstrated that YAP upregulation could decrease E-cadherin expression and increase N-cadherin expression and promote carcinogenesis and metastasis in human cholangiocarcinoma. Zhang et al [32] proposed that YAP1 potentiated TGFβ-driven Smad signalling to regulate the expressions of Snail, Slug, and Twist1, the important transcriptional regulators of EMT in the development of the atrioventricular cushion. Yuan Y et al [33] pointed out that inhibition of YAP suppressed the expressions of N-cadherin and snail, increased the expression of E-cadherin and inhibited the invasive ability of pancreatic cancer cells. Zeng G et al [34] also demonstrated that YAP1 promoted oral squamous cell carcinoma cells migration and invasion and enhanced the expressions of vimentin, snail and twist. Li et al [35] announced that TAZ promoted vimentin expression and reduced E-cadherin level in oral cancer. TEAD influenced EMT mainly through alterations in YAP and TAZ levels [36, 37]. These researches and our experimental data provide the theoretical basis that IKBKE promotes glioblastoma growth and EMT through IKBKE-dependent YAP1/TEAD2 activation.

Furthermore, previous studies have demonstrated IKBKE activated the NF-κB pathway [12, 13] and Akt related pathway [38] while the activation of NF-κB pathway and Akt related pathway was demonstrated to promote EMT [39–42] and expressions of some matrix metalloproteinases such as MMP2, MMP9 [13, 43]. So we did not rule out the effect of IKBKE on EMT through other pathways.

In conclusion, our research aimed that IKBKE can regulate glioma cell proliferation, migration, and invasion abilities *in vitro* and *in vivo*. More importantly, we testify that IKBKE accelerates EMT of glioblastoma cells via IKBKE-dependent YAP1/TEAD2 activation. IKBKE directly interacts with YAP1 and TEAD2 but the specific mechanism that IKBKE promotes YAP1/TEAD2 expression remains to be clarified. Our findings also serve as a reminder that IKBKE may be a potential new target for the treatment of malignant glioma.

## MATERIALS AND METHODS

### Cell culture

Seven human malignant glioma cell lines (LN-229,U87-MG,A172,U251,SnB19, LN-308 and H4) were purchased from the Institute of Biochemistry and Cell Biology (Shanghai, China) and were cultured in Dulbecco's modified Eagle's medium(DMEM,Gibco) with 10% FBS (Gibco). The cells were kept in an incubator at 37°C with an atmosphere of 5% CO_2_.

### Antibodies

IKBKE rabbit mAb (No.2905 WB 1:1000, IP 1:100), E-cadherin mouse mAb (No.14472 WB 1:1000 IHC 1:100), N-cadherin rabbit mAb (No.13116 WB 1:1000 IHC 1:100), MMP9 rabbit mAb (No.13667 WB 1:1000 IHC 1:100), Slug rabbit mAb (No.9585 WB 1:1000) and YAP1 mouse mAb (No. 12395 WB 1:1000 IHC 1:100 IP 1:100) were obtained from Cell Signaling Technology (USA). β-catenin rabbit polyclonal antibody (ab32572 WB 1:5000 IHC 1:100), Snail rabbit polyclonal antibody (ab180714 WB 1:1000 IHC 1:100), Vimentin rabbit polyclonal antibody (ab45939 WB 1:1000 IHC 1:10000), MMP2 rabbit polyclonal antibody (ab37150 WB 1:1000 IHC 1:100), Twist rabbit polyclonal antibody (ab49254 1:500), TEAD2 rabbit polyclonal antibody (ab83670 WB 1:500) and IKBKE rabbit polyclonal antibody (ab7891 IHC 1:100) were purchased from Abcam (UK). TEAD2 rabbit polyclonal antibody (sc-67115 IP 1:50) were from Santa Cruz (USA). GADPH mouse mAb (TA309157 WB 1:2000) was obtained from ZSGB-BIO (China).

### Total RNA extraction and real-time RT-PCR

Total RNA was extracted with TRIzol (Invitrogen, USA) after transfection for 48h; reverse transcription and real-time PCR were finished using GoScriptTM Reverse Transcription System and GoTaq qPCR Master Mix obtained from Promega (USA) according to the supplier's protocol. All primers were synthesized by GENEWIZ (USA). The nucleotide sequences of the primers are as follows: GAPDH: 5**′**-GGAGCGAGATCCCTCCAAAAT-3**′** and 5**′**-GGCTGTTGTCATACTTCTCATGG-3**′**; YAP1: 5**′**-TAG CCCTGCGTAGCCAGTTA-3**′** and 5**′**- TCATGCTT AGTCCACTGTCTGT-3**′**; TEAD2: 5**′**-GCCTCCGAGAG CTATATGATCG-3**′** and 5**′**- TCACTCCGTAGAAGCC ACCA-3**′**; E-cadherin: 5**′**-ATTTTTCCCTCGACACC CGAT-3**′** and 5′-TCCCAGGCGTAGACCAAGA-3**′**; N-cadherin: 5**′**-TGCGGTACAGTGTAACTGGG-3**′** and 5**′**-GAAACCGGGCTATCTGCTCG-3**′**; β-catenin: 5**′**-CG ACCTGGAAAACGCCATCA-3**′** and 5**′**-CCTATGCAGGG GTGGTCAAC-3**′**; and vimentin: 5**′**-TGCCGTTGA AGCTGCTAACTA-3**′** and 5**′**-CCAGAGGGAGTGAATCC AGATTA-3**′**. Each sample was taken in triplicate. GAPDH was used as an internal reference and the 2-ΔΔCT method was used to analyse PCR results.

### Transfections of lentiviral vectors with IKBKE shRNA and siRNA of YAP1, TEAD2 and plasmids of IKBKE, YAP1 and TEAD2

IKBKE shRNA was selected according to a previous article [13]. We constructed an IKBKE shRNA lentiviral vector (Shanghai GeneChem, Shanghai, China) using the most effective sequences shRNA: 5**′**-GCATCATCGAACGGCTAAATA-3**′**. A GFP scrambled lentiviral vector with sequences 5**′**-TTCTCCGAACGTGTCACGTTTC-3**′** was used as the negative control. The shRNA were transfected according to the manufacturer's instructions. To steadily knock down IKBKE, transfected cells were treated by 3 μg/mL puromycin. The designs of YAP1-siRNAs and TEAD2-siRNAs were from Ribobio (Guangzhou China), and the transfection process was performed according to the manufacturer's instructions. The plasmids of IKBKE, YAP1 and empty vector were purchased from addgene (USA) and TEAD2 and empty vector plasmids was from Hanheng biology (Wuhan, China).

### Wound healing cell migration assays and transwell cell invasion assay

Cell migration and invasion assays were carried out as described previously [44].

### Clone formation assay

U87-MG and LN-229 cells were seeded in six-well plates (2×103/well) divided into two groups as scrambled group and IKBKE-shRNA group. Growth medium was changed every 6 days. After 10 days, cells were fixed in 4% paraformaldehyde for 15min and stained with crystal violet for 30min. Colonies were photographed.

### Western blot analysis

Total cell lysate was prepared as described previously [13]. After denaturation, the proteins were separated by gel electrophoresis using 10% or 12% SDS-PAGE and electrotransferred to PVDF membranes (Millipore, Billerica, USA). After being washed three times by TBST, the membrane was blocked by 5% BSA for 1 hour at 37°C. Then, first antibodies were incubated overnight at 4°C. The membrane was again washed three times with TBST before incubation with a secondary antibody (goat anti-rabbit/mouse IgG 1:2000) for 1 hour at room temperature and was washed a third time with TBST. The proteins were detected by the G:BOX (Syngene Company, UK) using Chemiluminescent HRP Substrate (Millipore USA).

### Immunofluorescence

Different cells were seeded onto cover slips in a 12-well plate overnight. Cells were washed with PBS three times and fixed with 4% paraformaldehyde for 20 min. Then, they were permeabilized with 0.1% Triton X-100 for 15 min (except membrane antigen) and blocked in 5% BSA at room temperature for 30 min. They were then incubated with primary antibody at 4°C overnight. After being rewarmed for 1 hour, samples were washed with PBS three times and incubated with specific secondary antibodies for 1 hour at 37°C. After washing three times with PBS, the nuclei were stained with DAPI for 5 min at room temperature. Immunofluorescence was observed using fluorescence microscopy (Olympus, Japan).

### Co-immunoprecipitation (co-IP)

Co-immunoprecipitation was carried out as described previously [45].

### Immunohistochemical staining

Paraffin-embedded tumours were sectioned and dewaxed. After antigen retrieval (treated in 10 mmol/L citrate buffer for 20 minutes at 95°C), sections were cleared of endogenous peroxidase activity by incubation with 3% H_2_O_2_ for 15 min and blocked with 5% BSA for 30 min at 37°C. They were then incubated with primary antibodies at 4°C overnight. The next day, after rewarming for 1 hour, sections were incubated with biotinylated secondary antibodies for 1 hour at 37°C. They were then incubated with ABC-peroxidase for 1 hour before sections were coloured using a DAB Kit (ZSGB-BIO, China) and counterstained with haematoxylin. After dehydration, sections were examined using a light microscope.

### Animal studies

The tumour subcutaneous experiments method was carried out as described previously [13]. For the nude mouse tumour intracranial model, U87-MG with scrambled lentivirus and IKBKE shRNA lentivirus were respectively co-infected with a luciferase-expressing lentivirus. Then, the U87-MG cells were injected intracranially into 4-week-old BALB/c-nu mice. After 7 days, the tumours were measured by luminescence imaging using an IVIS Lumina Imaging System (Xenogen). During animal experiments, we strictly followed internal biosafety and bioethics guidelines.

### Statistical analysis

All data were repeated at least three times. Quantitative data are shown as the mean ± standard deviation (SD). We used SPSS software (version 16.0) for the statistical analyses and *P* < 0.05 was considered statistically significant.

## SUPPLEMENTARY FIGURE


