# Supplementary material for: IKBKE regulates cell proliferation and epithelial–mesenchymal transition of human malignant glioma via the Hippo pathway
Source: Oncotarget. 2017 May 10;8(30):49502–14. doi: 10.18632/oncotarget.17738 (PMC5564784; doi:10.18632/oncotarget.17738)
Supplement: Supplementary file 1 [file oncotarget-08-49502-s001.pdf]

IKBKE regulates cell proliferation and epithelial–mesenchymal transition of human malignant glioma via the Hippo pathway

Supplementary Materials

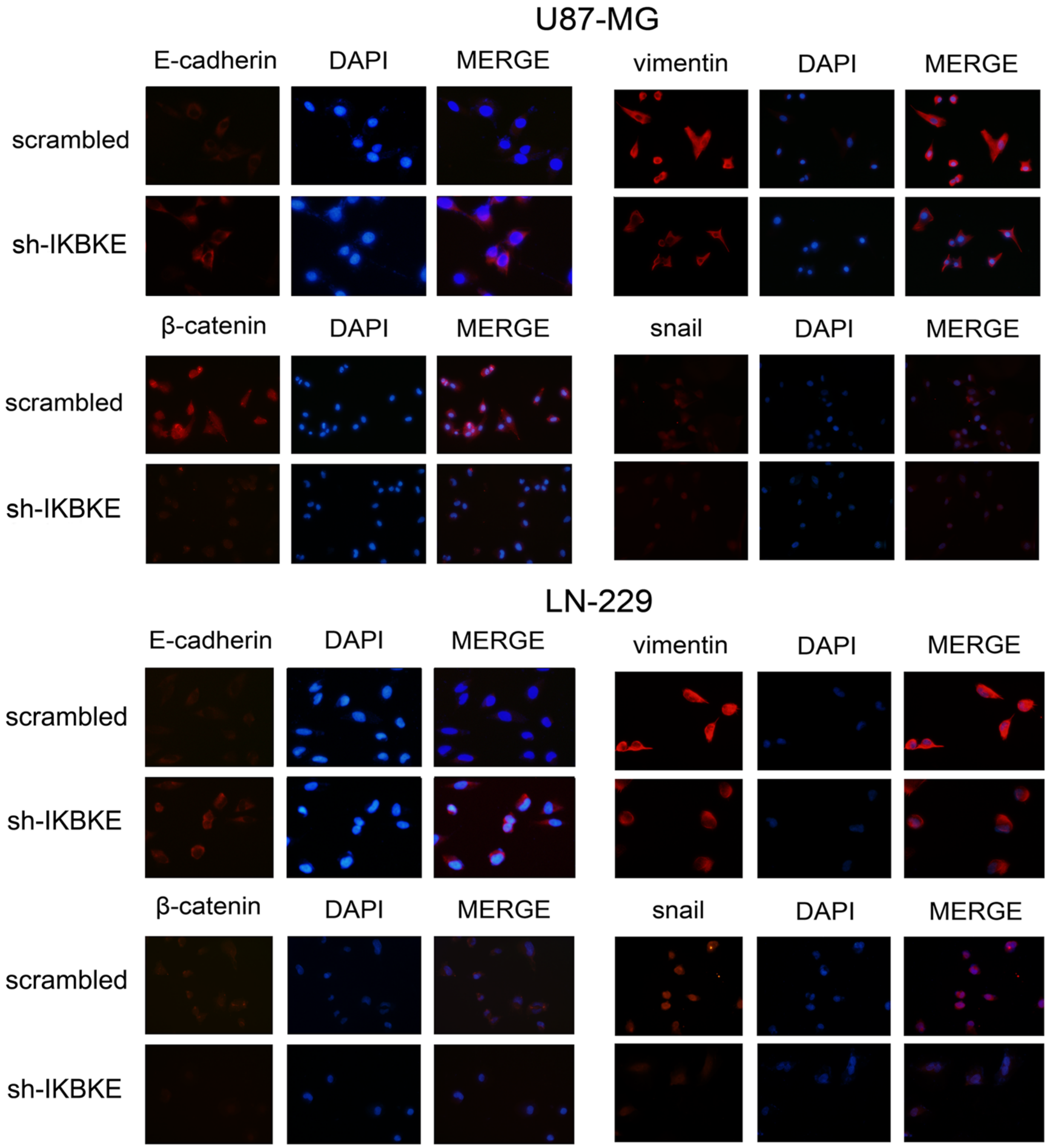

Supplemental Figure 1: The immunofluorescence analysis of E-cadherin, vimentin, β-catenin and snail after cells transfected with IKBKE-shRNA.
